# Is content of delusions in psychotic depression related to the risk of dementia?

**DOI:** 10.1192/j.eurpsy.2024.1315

**Published:** 2024-08-27

**Authors:** J. T. Coelho, B. Martins, A. Silva, C. Silveira, A. S. Machado

**Affiliations:** ^1^Department of Psychiatry and Mental Health; ^2^Department of Neurology, University Hospital Center of São João, Porto, Portugal

## Abstract

**Introduction:**

Some studies have shown that late-life depression is related to faster cognitive decline and may increase the risk of dementia.

Identifying risk and protective factors for dementia is essential to develop preventive interventions. Some literature has suggested that mood disorders (namely depression) are potential modifiable risk factors for dementia.

Thus, it is important to know clinical presentation of depression that is associated to dementia, as a manifestation of subclinical dementia or as a risk factor for neurocognitive disorders.

**Objectives:**

We aim to identify clinical characteristics related to dementia of inpatients admitted for first time due to depressive episode after 55 years old.

**Methods:**

Retrospective cohort study of inpatients admitted between January 1st 2010 and March 31st 2022 in a psychiatry inpatient unit of a tertiary hospital. Descriptive analysis of the results was performed using the SPSS software, version 26.0.

**Results:**

Our sample included 57 inpatients, 15,8% (n=9) with the diagnosis of dementia 5,2 (SD 5,6) years after admission. All of these patients presented a depressive episode with psychotic symptoms, namely delusion activity. In those with hallucinatory activity, no one developed dementia.

Interestingly, 33,3% of patients with dementia (n=3) presented with delusion of ruin, 55,6% (n=5) with delusion of prejudice/persecutory delusion and 66,7% (n=6) manifested delusion of ruin and/or prejudice.

We also found that 42,9% (n=3) of patients with dementia manifested Cotard delusion while this type of delusion was observed in 13,6% of patients without dementia (p=0,095).

**Conclusions:**

Our study has several limitations because is based on results of only one hospital, with a small sample size.

However, since depressive symptoms are potentially modifiable risk factors for dementia, future studies are essential to understand the mechanisms that link depression to cognitive decline as well as clinical characteristics that may constitute predictors of dementia.

**Disclosure of Interest:**

None Declared

